# Factors Associated with Visit-to-Visit Variability of Blood Pressure Measured as Part of Routine Clinical Care among Patients Attending Cardiology Outpatient Department of a Tertiary Care Centre in Northern Sri Lanka

**DOI:** 10.1155/2019/6450281

**Published:** 2019-12-05

**Authors:** Thirunavukarasu Kumanan, Vathulan Sujanitha, Mahesan Guruparan, Nadarajah Rajeshkannan

**Affiliations:** ^1^Department of Medicine, Faculty of Medicine, University of Jaffna, Jaffna, Sri Lanka; ^2^Department of Cardiology, Teaching Hospital-Jaffna, Jaffna, Sri Lanka; ^3^Civic Park Medical Centre, Sydney, NSW 2145, Australia

## Abstract

Visit-to-visit variability (VVV) is a relatively new concept in the hypertensive arena. Data regarding VVV are lacking in our region, and factors associated with VVV are rarely examined in previous studies. This study was conducted among 406 patients attended to the cardiology outpatient department of Teaching Hospital, Jaffna, Sri Lanka, in 2018 to assess the long-term variability of blood pressure (BP) by reviewing last six consecutive BP readings from the records retrospectively. Data regarding sociodemographic variables and behavioural factors such as medication adherence, physical activity, smoking, alcohol consumption, and relevant comorbidities were taken through an interviewer-administered questionnaire. Data were analysed by using SPSS version 25 and VVV of systolic blood pressure (SBP) matrix expressed as mean of SD and association were examined with various factors and VVV of SBP. SBP showed high VVV among the participants as expressed by mean of SD which was 13.06 ± 5.64. When comparing mean SD among the categories of different variables, female sex (*P*=0.023) and comorbidities such as diabetes mellitus (DM) (*P*=0.013), chronic kidney disease (CKD) (*P*=0.007), and risk of developing obstructive sleep apnoea (OSA) (*P*=0.04) showed significant variation. Medication adherence to prescribed hypertensive medication was a major issue even though significant association was not found with high VVV (*P*=0.536). The SD of SBP was then classified into high and low VVV groups by means of a cutoff point at the 50th percentile. Bivariate analysis by using Chi-squared test revealed comorbidities such as DM, CKD, and physical activity (*P*=0.044) were significantly associated with high VVV. Further multivariate regression analysis revealed that comorbidities such as DM and CKD have 1.561 times and 5.999 times more risk to show high variability, respectively. In conclusion, we recommend simple practical measures to achieve sustainable BP control among hypertensive patients with DM and CKD to minimize the VVV and improve their cardiovascular outcome.

## 1. Introduction

Blood pressure fluctuation is a well-known phenomenon among patients with hypertension known as visit-to-visit variability (VVV). In the past, VVV of blood pressure often dismissed as random fluctuation and is thought to be a limitation of measuring blood pressure at clinic settings [1]. One of the most interesting finding of hypertension research in the past ten years has been that the cardiovascular protective effects of antihypertensive drugs depend not only on the mean blood pressure value achieved during treatment but also on the consistency of blood pressure control between treatment visit [2].

VVV is an important independent prognostic marker for stroke, cardiovascular disease, and all-cause mortality [3–5]. The mechanism underlying the association between VVV of blood pressure and the outcomes are poorly understood. Blood pressure variability could be classified into short-term, midterm, and long-term variability. This study was performed among hypertensive patients (*n* = 406) to assess the long-term variability by looking into their outpatient clinic records. Short-term variability in blood pressure is affected by behavioural, emotional, and postural influences on cardiovascular physiology and cardiac rhythm [6, 7]. Arterial stiffness contributes to both short-term and long-term variability of blood pressure [8, 9].

Antihypertensive nonadherence would explain the adverse prognosis found in individuals with high VVV of blood pressure and may be a modifiable cause of VVV of blood pressure, and efforts to reduce nonadherence have the potential to decrease the VVV of blood pressure [10]. Additionally, it was found that chronic kidney disease is associated with more severe hypertension and high VVV of blood pressure [11], and also higher long-term blood pressure VVV is associated with a faster rate of cognitive decline among older adults [12].

As described above in the past, VVV is thought to be a nuisance factor and considered as not related to future risk of development of cardiovascular diseases. But recent studies primarily from Europe found strong association between higher levels of variability of blood pressure and increased risks of coronary heart disease and stroke, and evidences also revealed that VVV is not a random phenomenon [3, 13–15]. Association with external factors and VVV of blood pressure is rarely examined in studies. Though many studies are performed in Sri Lanka related to hypertension, to our knowledge, VVV of blood pressure and its association is not reported in the literature to date. This study aimed to determine the level of VVV in our population by using blood pressure data measured as a part of routine clinical care and also intended to identify associated factors of blood pressure variability in our population such as lifestyle factors, medication adherence, and comorbidities.

## 2. Materials and Methods

This cross-sectional study was conducted among 406 patients attended to the cardiology outpatient clinic of Teaching Hospital, Jaffna, Sri Lanka, in 2018. Patients with hypertension of more than one year duration who are 18 years of age and more were included in the study. Data were collected over the period of two months by using an interviewer-administered questionnaire. Last six consecutive BP recordings were retrieved after obtaining informed consent from participants. This study was evaluated and approved by the Ethics Review Committee, Faculty of Medicine, University of Jaffna, Sri Lanka.

### 2.1. Data Collection

#### 2.1.1. BP and VVV

Data regarding BP were retrospectively retrieved from medical records. BP was measured in office-based settings with an interval of 8 weeks by medical officers attached to the cardiology department with the conventional cuff method using a mercury sphygmomanometer. In this study, we used the standard deviation (SD) of BP of six visits as the main metric for VVV of SBP regardless of their being many other metrics available (coefficient of correlation, SD independent of the mean (SDIM)) as previous study found them to be equally significant. The SD of SBP was then classified into two groups by means of a cutoff point at median: values below the median were defined as low variability of SBP, and values equal to or greater than median are defined as high variability of SBP [16]. We also reported average real variability of SBP and diastolic blood pressure (DBP) which is calculated as the average of the absolute difference in blood pressure between visits [17]. For example, the first participant had six visits with the following systolic BP measurements: 150, 130, 120, 120, 120, and 120; hence, the ARV would be calculated as (|150 − 130|+|120 − 130|+|120 − 120|+|120 − 120|+|120 − 120|)/5=6 mm Hg.

#### 2.1.2. Covariates

Patient's clinic records were used to get information including year of diagnosis of hypertension and comorbidities such as DM, CKD, and hypertensive medication.

Quantity of alcohol was calculated in units by using the validated questionnaire used in different studies in Sri Lanka. Physical activity level was calculated using International Physical Activity Questionnaire (IPAQ score). Both questionnaires were used in many studies of Sri Lanka, and validity was established already.

Weight, height, neck circumference, and midarm circumference were done by standard weighing scale and nonstretchable plastic tape.

The neck circumference was measured in the midway of the neck, between the midcervical spine and midanterior neck using a nonstretchable plastic tape with the subjects standing upright. The midarm circumference of the upper arm is measured at the point of half way between the olecranon process of the ulna and the acromion process of scapula using the nonstretchable plastic tape.

Sleep apnoea risk was assessed using the STOP-Bang questionnaire. This questionnaire was translated in to Tamil and was retranslated to English to check that the meaning was not altered, and also consensus was obtained among three different physicians. Risk was categorised as low, medium, and high.

Medication adherence was classified as low, medium, and high adherence based on a standard modified medication adherence questionnaire. This questionnaire also was translated in to Tamil and was retranslated to English to check that the meaning was not altered, and also consensus was obtained among three different physicians.

*Statistical Analysis*. Statistical analysis was performed with IBM SPSS Statistics version 25 [17]. Participants' sociodemographic characteristics, lifestyle factors, adherence to antihypertensive medications, and other covariates were compared with low VVV of SBP and high VVV of SBP groups. Data were expressed as a percentage, mean ± SD, or median (and range). Bivariate analysis using the chi-squared test for categorical variables was used to determine statistically significant differences between variables. All significant variables were included in the multivariate regression test. Multivariable analysis was performed to calculate the *β*-coefficient (standard error) and adjusted exp *β* (odds ratio (OR), 95.0% confidence interval (CI)).

We also examined comparison of the mean of SD of SBP between groups of different variables. Comparisons of the mean values between three or four groups were analysed by the one-way analysis of variance.

## 3. Results and Discussion

### 3.1. Results

#### 3.1.1. Baseline Characteristics

Most of the participants were males (62.6%) and belong to 50–64 age group (82.3%). Majority studied up to secondary education (78.6%), and 71.2% of the participants were housewives or unemployed. Interestingly, most of them belong to poorest income group (63.5%) and 34.4% reported as middle income.  Table 1 shows sociodemographic characteristics of patients with hypertension who attended the cardiology outpatient department.

#### 3.1.2. Lifestyle and Behavioural Characteristics

Considerable amount of the participants (26.6% CI: 22.5–31.1) were noted to have insufficient (sedentary) physical activity level and 14.3% (CI: 11.1–17.9) were obese. As shown in Table 2, majority revealed no smoking history 68.2%(CI: 63.6–72.6), and only 2.7% patients reported that they were currently taking alcohol, but all reported taking within recommended level. Most of the participants (56.2%) diagnosed to have hypertension for more than 5 years, and none of them reported high medication adherence to prescribed hypertensive medication(s), and 22.7% (CI: 18.8–26.9) reported low adherence to medication.

When considering the associated comorbidities (Table 3), majority of the hypertensive patients had coronary artery disease (CAD) (92.9%) as expected in the context of the study setting at a cardiology outpatient department, and 47.5% (CI: 42.7–52.5%) had DM as an associated comorbidity. Only 4.2% hypertensive patients had confirmed CKD; however, 265 patients' (65.3%), who had serum creatinine levels, revealed that 118 (29.1%) of them had above normal levels. Risk of developing OSA was assessed by internationally well-known questionnaire (STOP-Bang Questionnaire), and 65.3% (60.5%–69.8%) were found to have high risk to develop OSA.

**Figure 1:**
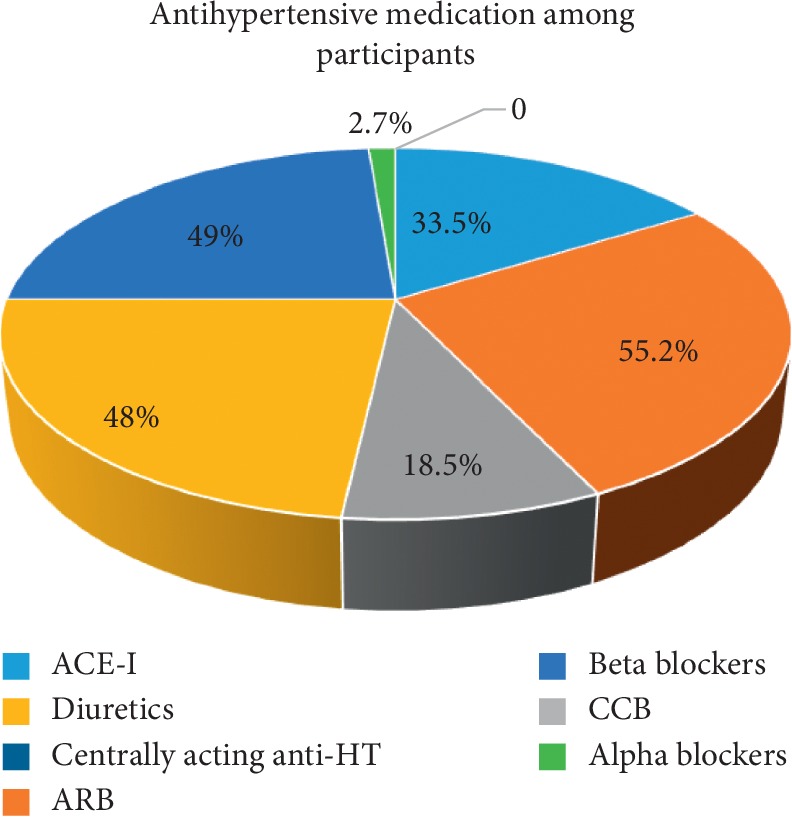
Antihypertensive medications among participants.

When considering the antihypertensive medications among the participants (Figure 1), commonly prescribed hypertensive medication class was angiotensin receptor blockers (ARB) (55.2%) followed by beta blockers (49%), diuretics (48%), angiotensin converting enzyme inhibitors (ACE-I) (33.5%), and calcium channel blockers (CCB) (18.5%). Among the diuretics commonly prescribed diuretics were thiazides (*n* = 34).

#### 3.1.3. Outcome Variables (VVV) and Associated Factors

**Figure 2:**
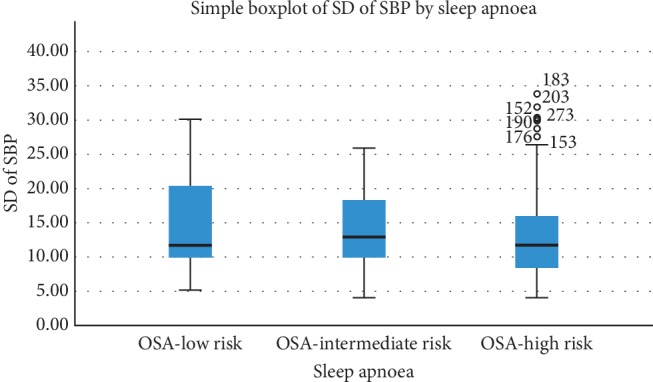
Variation of SD by sleep apnoea.

The average means of SBP and DBP were 127.4 ± 8.62 mm Hg and 78.40 ± 3.64 mm Hg, respectively and were obtained from 6 measurements. The mean average real variability (ARV) of SBP was 14.10 mm Hg ± 7.79. The range of ARV of SBP was 2–48 mm Hg. The average mean of SD was 13.06 ± 5.64, and we examined mean SD comparison among the categories of different variables and found that female sex (*P*=0.023), DM (*P*=0.013), CKD (*P*=0.007), and risk of developing OSA (*P*=0.04) showed significant variation. No other variables showed significant variation.  Figure 2 shows variation of SD among the different categories of risk of developing OSA.

The SD range of SBP was 4.08–33.86 mm Hg, and a median value of 11.69 mm Hg was used as a cutoff point to further classify participants into two groups; the group with low VVV of SBP and the group with high VVV of SBP.

The characteristics of the patients in each study group are summarized in Table 4. The demographics, socioeconomic status, current alcohol drinking, smoking status, types of antihypertensive being used, body mass index, and medication adherence did not differ significantly between the low VVV group and the high VVV group. Except DM and CKD, no other comorbidities were significantly associated with high VVV.

*Multivariable Analysis*. The associations between study variables and VVV of SBP were further analysed in a multivariate regression logistic test, and significance was consistent for associated DM (*P*=0.033) and CKD (*P*=0.019). Physical activity has become nonsignificant (*P*=0.098). After adjustment for covariates such as age, sex, economic status, education, and occupation, DM and CKD was significantly associated with high VVV of SBP (Table 5).

1.561 times and 5.999 times more risk to show high variability is shown when having DM and CKD as comorbidities, respectively.

### 3.2. Discussion

Many studies have been carried out recently on blood pressure VVV as it is emerging as an important independent risk factor for cardiovascular events and deaths. Rothwell et al. showed almost a decade back by a series of publications at Lancet and Lancet neurology that VVV of blood pressure is a strong risk factor for stroke, independent of mean blood pressure. Subsequently, VVV was identified as a novel risk factor for cardiovascular disease [3, 18, 19].

A study was carried out in the past by using blood pressure measurements which were taken as part of the routine outpatient care over a median of 2.8 years was analysed and concluded as intraindividual VVV was not a random phenomenon [20]. Also, a previous study among diabetics revealed that patients with a low SD of SBP of <5 mmHg had the lowest risks of CVD and all-cause mortality compared with patients with a SD of SBP of ≥10 mmHg who had significantly higher risks [21]. Our study found that VVV of SBP was high, SD was 13.06 ± 5.64, and the mean average real variability of SBP was 14.10 mm Hg ± 7.79.

Another study revealed being older, female gender, and certain comorbid conditions significantly raised VVV [22]. This study also found that female sex has significant association (*P*=0.023) with high VVV while comparing means of SD with counterpart. No other sociodemographic variables showed significant variation. A previous study also found sodium intake was significantly higher in the high VVV group than in the low VVV group [16], but we have not assessed the sodium intake in our population, and it is a potential limitation of our study.

Several studies found that the VVV was associated with progression of vascular disease such as dementia [23], cognitive dysfunction [23], and development and progression of diabetic kidney diseases [24]. In this study, among the comorbidities, DM (*P*=0.013) and CKD (*P*=0.007) have showed significant variation when comparing mean standard deviation. Further analysis (regression analysis) by comparing low VVV and high variability found that having DM and CKD as comorbidities have 1.561 times and 5.999 times more risk to show high variability, respectively. This fact is well supported by the study carried out among type 2 diabetics which concluded that VVV in SBP could be a novel risk factor for development and progression of nephropathy [21]. In the study participants, 47.5% (CI: 42.7–52.5%) had DM. Even though only 4.2% patients had diagnosed CKD, retrieved data from the records of 265 patients' (65.3%) last serum creatinine level revealed that 118 (29.1%) had above normal values. In 2018, Viazzi et al. reported controversies in identifying target BP in managing renal patients and concluded that targeting systolic blood pressure around 130 mmHg would be suffice and consider further lowering in the presence of overt proteinuria. Adopting this target could therefore results in both renal and cardiovascular protection [25]. It was recommended further that estimating albuminuria could be a guide for optimal therapeutic regime among hypertensive diabetics [26]. Same authors also showed long-term variability of blood pressure increases the risk of developing CKD [27]. We believe that there is an urgent need to give more attention in diagnosing CKD early with standard investigations in the study setting. Furthermore, it is also very important to control VVV of SBP in order to prevent further progression of CKD and prevent future risks of CVD and all-cause mortality.

We used STOP-Bang questionnaire to assess the risk of developing OSA, and 65.3% (60.5%–69.8%) found to have high risk to develop OSA. We also compared mean of SD of SBP among low-, medium-, and high-risk OSA groups (Figure 2) which showed significant association with VVV (*P*=0.04). But we were unable to establish a significant association between risks of developing OSA with high variability during further analysis, and the reason might be that the STOP-bang questionnaire was not validated to our own population. But a study on sleep apnoea and VVV concluded that OSA is associated with abnormal visit-to-visit variability, and sympathetic activation seems to be related in some way [28]. A screening and detailed evaluation of OSA is not usually done in this part of the world. The lack of awareness about OSA and unavailability of evaluation tools is the most likely reason. So, we recommend to do further studies on this subject and treating physicians should consider to assess sleep apnoea if VVV of SBP is high.

A study at a cardiac rehabilitation setting measured SBP and DBP before exercise training at each visit and determined VVV in BP expressed as the standard deviation of the average BP. Patients who had uncontrolled BP at baseline and who did not change their antihypertensive drugs throughout the study period showed a significant reduction of both SBP and DBP after 3 months. Patients who did not change their antihypertensive drugs were divided into high and low VVV in the SBP groups according to the average value of VVV in SBP or DBP and found that VVV in SBP and DBP in the 1st month was significantly decreased than the 3rd month in both groups [29]. Our study revealed considerable amount of the participants (26.6% CI: 22.5–31.1) had insufficient (sedentary) physical activity level, and 20.4% (16.7–24.6) reported as moderately active. Physical activity (*P*=0.044) showed significant association with high variability in bivariate analysis and multivariate analysis did not show significant association (*P*=0.098). But we could argue the role of physical activity in control of VVV of SBP and its importance to encourage patients to involve more physical activity.

It is reasonable to predict class of antihypertensive drugs also may have impact in the long-term variability. A study evaluated whether the antihypertensive medication class differentially affected blood pressure VVV among hypertensive individuals in clinical real-world setting as well as the association between VVV and characteristics. It showed diuretics significantly lowered VVV and alpha/beta blockers resulted in highest VVV [14, 19]. But this study failed to show any significant association between any class of hypertensive medication and SBP VVV. Specifically beta blockers (0.077) and diuretics (*P*=0.151) not showed significant difference with VVV of SBP.

Similarly a study on medication adherence and VVV of systolic blood pressure in African Americans with chronic kidney disease (CKD) has found lower medication adherence is associated with higher systolic VVV of blood pressure in CKD patients. Efforts to improve medication adherence in this population may reduce systolic VVV of blood pressure [30, 31]. We examined the VVV of SBP among the low-adherence and the medium-adherence group. The statistical analysis showed no significant association (*P*=0.536) between the two groups. The reasons could be medication adherence is low regardless of different categories of VVV and also be due to the adherence questionnaire is not validated among our population. None of the participants of the study reported high adherence, and 22.7% (CI: 18.8–26.9) reported low adherence to medication. A previous study in the same setting also revealed poor medication adherence was prevalent due to several reasons [31]. Addressing medication adherence is an important task of treating physicians in these circumstances.

As ample evidence available for VVV and all-cause mortality [32], it could be an important area that the treating physician to work on their hypertensive patients.

## 4. Conclusion

This study clearly demonstrates that VVV of BP is high among our population and high VVV independently associated with comorbidities DM and CKD. Hence, the clinicians should not consider the adequacy of blood pressure control at a single visit as it is of marginal clinical importance. VVV of BP is considerably high among the study population, and addressing this may reduce patients' cardiovascular morbidity and mortality in poor resource setting. A most stringent BP control is recommended in particular among hypertensive patients with comorbidities of DM and CKD. As medication adherence plays a key role in treating hypertensives, clinicians should adhere to simple prescription pattern to ensure compliance and consequently minimize VVV. Identifying and treating sleep apnoea would also have a great impact on VVV, and it is always desirable to conduct studies related to sleep apnoea in selected patients. Encouraging physical activity could be an important strategy to ameliorate VVV of SBP among hypertensives. In conclusion, VVV of BP is an important target for the treating physician to reduce the cardiovascular and renal outcome of diabetic patients with hypertension.

## Figures and Tables

**Table 1:** Sociodemographic characteristics patients with hypertension (*n* = 406).

Variable	Categories	Number	Percentage
Age	40–49	13	3.2
	50–64	334	82.3
	65 and above	59	14.5
Sex	Male	254	62.6
	Female	152	37.4
Highest education achieved	No formal education	6	1.5
	Primary	63	15.5
	Secondary	319	78.6
	Tertiary	131	4.4
Present occupation	Senior officials and managers	18	1.0
	Professionals	8	2.0
	Technical and associate professionals	1	0.2
	Clerks	2	0.5
	Sales and service workers	23	5.7
	Skilled agricultural and fishery workers	17	4.2
	Craft and related trade workers	11	2.7
	Plant and machine operators and assemblers	8	2.0
	Elementary occupations	42	10.3
	Unemployed/housewife	289	71.2
	Others	1	0.2
Monthly family income	Poorest (less than Rs. 16,162)	258	63.5
	Middle income (Rs. 16,163–Rs. 5749)	138	34.0
	Rich (>Rs. 57,500)	10	2.5

**Table 2:** Behavioural characteristics of patients with hypertension (*n* = 406).

Variable	Categories	Number	Percentage with confidence interval (CI)
Smoking status	Never	277	68.2 (63.6–72.6)
	Exsmoker	121	29.8 (25.5–34.4)
	Current smoker	8	2.0 (0.3–2.4)
Consumption of alcohol status	Exdrinker	97	23.9 (19.9–28.2)
	Nondrinker	298	73.4 (68.9–77.5)
	Current drinker	11	2.7 (1.4–4.7)
Physical activity	Insufficiently active (sedentary)	108	26.6 (22.5–31.1)
	Moderately active	83	20.4 (16.7–24.6)
	HEPA active	215	53.0 (48.1–57.8)
BMI	Underweight	9	2.2 (1.1–4.0)
	Normal weight	206	50.7 (45.9–55.6)
	Over weight	133	32.8 (28.3–37.4)
	Obesity	58	14.3 (11.1–17.9)
Years from diagnosis	Newly diagnosed (<5 years)	178	43.8 (39.1–48.7)
	≥5 years	228	56.2 (51.3–60.9)
Medication adherence	Low adherence	92	22.7 (18.8–26.9)
	Medium adherence	314	77.3 (73.1–81.2)

**Table 3:** VVV and associated comorbidities.

Condition	No	Percentage with 95%: CI
Diabetes mellitus (DM)	193	47.5 (42.7–52.4)
Ischaemic heart disease (IHD)	377	92.9 (90.0–95.1)
Heart failure	5	1.2 (0.5–2.7)
Chronic kidney disease (CKD)	17	4.2 (2.5–6.5)
Cerebrovascular accident (CVA)	21	5.5 (3.3–7.7)
Sleep apnoea		
OSA: low risk	53	13.1 (10.0–16.6)
OSA: intermediate risk	88	21.7 (17.9–25.9)
OSA: high risk	265	65.3 (60.5–69.8)

**Table 4:** Characteristics of participants based on the level of visit-to-visit variability (VVV) of systolic blood pressure (SBP).

Characteristics	VVV		*P* value
	Low variability (188)	High variability (218)	
Sex			
Male	125	129	0.129
Female	63	89	
Income			
Poorest	118	140	0.674
Middle Income	64	74	
Rich	6	4	
Age			
40–49	5	8	0.656
50–64	153	181	
65 and above	30	29	
Education			
No education	2	4	0.733
Primary	26	37	
Secondary	151	168	
Tertiary	9	9	
Alcohol			
Current drinkers	6	5	0.635
Smoking			
Current smokers	6	2	0.256
Physical activity			
Insufficiently active	42	66	0.044^*∗∗*^
Moderately active	34	49	
HEPA active	112	103	
Medication adherence			
Low adherence	40	52	0.536
Medium adherence	148	166	
Years from diagnosis			
Less than 5 years	81	97	0.775
≥5 years	107	121	
BMI			
Underweight	7	2	0.270
Normal weight	92	114	
Over weight	63	70	
Obesity	26	32	
Comorbidities			
DM	76	117	0.008^*∗∗*^
CKD	2	15	0.004^*∗∗*^
Medication			
Beta blockers	88	111	0.409

Bivariate analysis by using the Chi-squared test revealed DM (*P*=0.008), CKD (*P*=0.004), and physical activity (*P*=0.044) were significantly associated with high variability.

**Table 5:** Odds ratios of comorbidities for high visit-to-visit variability patients.

Comorbidities	AOR	CI	*P* value
DM	1.561	1.036–2.353	0.033
CKD	5.999	1.336–26.929	0.019

## Data Availability

Data can be provided on request.

## Abbreviations

VVV: Visit-to-visit variability
SBP: Systolic blood pressure
BP: Blood pressure
SD: Standard deviation
ARV: Average real variability
DM: Diabetes mellitus
IHD: Ischaemic heart disease
CKD: Chronic kidney disease
BMI: Body mass index
CI: Confidence interval
AOR: Adjusted odds ratio.
